# Suitability and Comparison of Questionnaires Assessing Virtual Reality-Induced Symptoms and Effects and User Experience in Virtual Environments

**DOI:** 10.3390/s21041185

**Published:** 2021-02-08

**Authors:** Andrej Somrak, Matevž Pogačnik, Jože Guna

**Affiliations:** Faculty of Electrical Engineering, University of Ljubljana, Tržaška CESTA 25, 1000 Ljubljana, Slovenia; matevz.pogacnik@fe.uni-lj.si (M.P.); joze.guna@fe.uni-lj.si (J.G.)

**Keywords:** virtual reality, VR Sickness, VRISE, user experience, user study, suitability assessment

## Abstract

Although virtual reality (VR) has already achieved technological maturity, there are still some significant drawbacks for technology acceptance and broader user adoption, presenting research challenges. Thus, there is a need for standard, reliable, and quick assessment tools for Virtual Reality-Induced Symptoms and Effects (VRISE) and user experience in VR Assessing VRISE and user experience could be time consuming, especially when using objective physiological measures. In this study, we have reviewed, compared, and performed a suitability assessment of existing standard measures for evaluating VRISE and user experience in VR We have developed a first-person VR game with different scenes and different conditions. For assessing VRISE symptoms, we have used the Simulator Sickness Questionnaire (SSQ) and Fast Motion Sickness Score (FMS). For assessing user experience, we have used the short version of the User Experience Questionnaire (UEQ-S). We have also used a novel Virtual Reality Neuroscience Questionnaire (VRNQ) for assessing VRISE and user experience aspects. The result has shown that FMS and VRNQ (VRISE section) are suitable for quick assessment of VRISE and that VRNQ (User experience section) is suitable for assessing user experience. The advantage of FMS and VRNQ questionnaires is that they are shorter to fulfill and easier to understand. FMS also enables to record the VRISE levels during the virtual experience and thus capturing its trend over time. Another advantage of the VRNQ is that it also provides the minimum and parsimonious cut-offs to appraise the suitability of VR software, which we have confirmed in our study to be adequate.

## 1. Introduction

Virtual Reality (VR) technologies have achieved technological maturity and are no longer included in the Gartner’s Hype cycle of emerging technologies since 2018 [1]. Still, the health-oriented side effects are still present, which can negatively impact the broader technology adoption. In the last five years, a lot of VR devices and technology solutions were introduced. Plenty of different VR applications, dedicated for home or personal use and professional use, have been released. There are several interaction and locomotion techniques, which can also affect the impact of technology on users. Thus, there is a need for standard, reliable and quick assessment tools that should be developed, especially for VR Their aim should be focused to assess and compare different technology solutions and to assess the impact of different interaction and locomotion techniques.

### 1.1. VRISE Assesment and Evaluation

The presence of virtual reality-induced symptoms and effects (VRISE) can be evaluated by subjective self-reports or by objective physiological measures. The most used subjective questionnaire for assessing VRISE is the Simulator Sickness Questionnaire (SSQ) [2], which became the standard measure to assess VRISE in VR SSQ has been criticized for its shortcomings in some works [3,4]. In fact, SSQ was developed in 1993 in the context of simulation to assess simulator sickness in flight simulators, based on U.S. Navy pilot experimental data, for which the SSQ was designed and validated. Being the standard measure and most commonly one used in VR research, the SSQ questionnaire is the proper choice for new assessment tools to be compared and tested for suitability to assess VRISE in VR applications.

An alternative to the SSQ questionnaire are single-item nausea or motion sickness questionnaires, which are appropriable for quick assessments of VRISE. They share a common approach, asking participants to rate on a single scale feelings of either nausea or discomfort. The Subjective Units of Distress Scale (SUDS) [5] is one of such questionnaires, which has been utilized and verified in previous studies [6,7]. The SUDS Questionnaire can be utilized as a measure of both physical and emotional discomfort. It is frequently used in exposure treatment, research on anxiety disorders and phobias and, exposure treatment, and panic disorder. Visual Analogue Scale (VAS) is another single-item scale, which was used in previous studies [8,9] to assess subjective VRISE levels. Another widely used questionnaire is The Nausea profile [10], which was designed for medical use for the evaluation of the characteristics of nausea across individuals and situations with the goal of obtaining a more in-depth description of what patients are experiencing when they report the feeling of nausea. It consists of 17 items which are rated on a scale of 0 (not at all) to 9 (severely).

Another subjective questionnaire, which was utilized in this study is the Fast Motion Sickness Score (FMS) [11]. The FMS is a single-item verbal rating scale where participants gave a score from 0 (no sickness at all) to 20 (frank sickness) to evaluate the level of their sickness they felt in the (virtual) experience. In contrast to the SSQ Questionnaire, where VRISE levels are measured before or after the virtual experience, FMS also allows to measure participant’s VRISE levels during the experience and thus capturing its trend over time.

### 1.2. User Experience Assesment and Evaluation

Besides health-oriented side effects that come with VR usage, the quality of user experience also has an important impact on the user technical adoption and success of VR technology. Assessing user experience in VR is possible with several questionnaires, but none is widely adopted yet. Some studies used the User Experience Questionnaire (UEQ) [7] and the meCUE questionnaires [12], which are based on the components of the User Experience model. The UEQ is used to measure the user experience of interactive products and gives insights into more complex aspects of user experience. For assessing user experience, the Game Experience Questionnaire (GEQ) [13] was also utilized in some previous studies in VR [14,15]. The GEQ is widely applied by game researchers to a broad spectrum of game genres. The Game Experience Questionnaire has a modular structure and consists of: (1) the core questionnaire, (2) the social presence module, and (3) the post-game module. In addition to these modules, a concise in-game version of the GEQ was developed. In our study, we have used the short version of the User Experience Questionnaire (UEQ-S) [16], which allows a quick assessment of user experience. UEQ-S measures two dimensions (quality aspects) of user experience. Pragmatic quality describes interaction qualities related to the tasks or goals the user aims to reach when using the product, and hedonic quality, which does not relate to tasks and goals but describe aspects related to pleasure or fun while using the product.

### 1.3. Virtual Reality Neuroscience Questionnaire

A promising novel Virtual Reality Neuroscience Questionnaire (VRNQ) [17], which we have validated for suitability in this study, is dedicated for usage in VR applications. It assesses and reports overall VR experience in four sections (VRISE, user experience, in-game assistance, and game mechanics). Compared to the SSQ and FMS questionnaires, it also assesses software attributes, not just only symptoms pertinent to VRISE. It also has an advantage in that it provides the minimum and parsimonious cut-offs to appraise the suitability of VR software. The minimum cut-offs indicate the lowest acceptable quality of VR software, while the parsimonious cut-offs indicate more robust VR software suitability.

### 1.4. The Results of the Study and Key Contributions

This paper presents the results of a user study of the suitability and comparison of standard questionnaires assessing Virtual Reality-Induced Symptoms and Effects (VRISE) and user experience in virtual environments. We have developed a first-person virtual reality (VR) game with different scenes (forest, ancient desert, and village) and different conditions (high action and low action mode, with or without head-centric rest-frames). Rest-frames are a basis of Rest Frame Hypothesis [18], an alternate theory of why VRISE does occur, where the emphasis is on the role of spatial-perceptual references affected by rest-frames. There are two types of rest-frames: egocentric and allocentric rest-frames. Head-centric rest-frames are of egocentric type that are centered on the player, specifically being centered and stationary relative to the player’s head.

Moderate to very strong correlations between FMS, VRNQ (VRISE subscale), and SSQ results indicate that FMS and VRNQ (VRISE subscale) are suitable for assessing VRISE levels. We have also found moderate to very strong correlations between UEQ-S and VRNQ (user experience sub-scale) scores, which indicates that the VRNQ (user experience sub-scale) is suitable for assessing user experience in VR The main advantage of FMS and VRNQ questionnaires is that they are shorter to fulfill and easier to understand by the respondents. FMS also enables to record the VRISE levels during the virtual experience and capture its time course. Another advantage of VRNQ is that it is dedicated for usage in virtual reality applications, and it also provides the minimum and parsimonious cut-offs to appraise the suitability of VR software. As VRNQ also enables us to appraise the suitability of VR software, we have also assessed the suitability of our game.

The key contributions of this paper are:

- Shown moderate to a strong correlation between results of a VRISE questionnaires (SSQ, FMS, and VRNQ—VRISE subscale), which indicates that FMS and VRNQ—VRISE subscale are suitable for quick assessment of VRISE levels;
- Shown moderate to a strong correlation between results of a UEQ-S and VRNQ—User experience subscale, which indicates that VRNQ—User experience subscale is suitable for the assessment of user experience VR;
- Shown adequacy of measuring VR software suitability with the VRNQ Questionnaire;
- Review, comparison, and suitability assessment of existing standard measures for evaluating VRISE and user experience in VR.

## 2. Background and Related Works

In this section, we review relevant related works. We have reviewed works related to the research of assessing VRISE and user experience in virtual environments and comparing different measures.

Factors that impact VRISE include individual, device, and task differences. The individual factors include gender, age, illness, and positioning [19]. It was being argued by LaViola [20] that women appear to be more susceptible to VRISE than men. That could be due to the wider field of view (FOV) that women have (comparing to men). A wide FOV increases the likelihood of flicker perception [20], which increases women’s susceptibility to VRISE. The reason for gender difference might also be influenced by the menstrual cycle and differences in postural stability between men and women [21]. In the study by Chang et al. [22], inconsistent results have been drawn across included studies in the review article. Stanney et al. [23] found out that interpupillary distance (IPD) non-fit was the primary driver of gender differences in VRISE, with motion sickness susceptibility identified as a secondary driver.

Age also seems to be an important factor that influences VRISE, but the results from the studies have shown mixed and opposite results [22]. For this reason, the authors [22] concluded that more studies are needed to explain the age effect on VRISE since other variables such as motion sickness susceptibility and prior VR experiences are also closely related to age. In a review paper by Saredakis et al. [24], they have found out that older participants (mean age ≥ 35 years) scored significantly lower total SSQ means than younger participants. However, these findings were based on a small evidence base, since there were limited number of studies that included older participates.

The task factors include control and duration [19]. Longer exposure times to VR results in increased VRISE, which requires longer adaptation periods. Using brief exposures to VR is one of the methods to improve the speed of the adaptation. By having users increase their exposure times gradually, they can adapt to the VR [20]. In the study by Howarth and Hodder [25], they have immersed 70 participants on ten occasions. The participants were exposed every day, every two days, and so on to every seven days. All sets of participant groups reported a marked reduction in the prevalence and severity of VRISE. They have found out that the number of exposures is a more important factor than the time between them. The limitation of the adaptation/habituation method is the significant commitment from VR users and the uncertainty of how durable the benefits of the adaptation may be [21]. Another problem is that the more adapted the individuals become to the virtual world, the more likely they are maladapted to the real world, which could have long-lasting after-effects [26].

In our previous study [7], we presented the results of a user study of the effects of virtual reality technology on VRISE and user experience aspects and evaluated the suitability of the Subjective Units of Distress Scale (SUDS) questionnaire for assessing VRISE. SUDS is a single-item scale, similar to FMS, where participants give a score on a single-scale based on the level of their anxiety or fear they felt in the (virtual) experience. The results have shown that SUDS is suitable for quick assessment of VRISE levels. In the study, we have used the standard User Experience Questionnaire (UEQ) [27] to assess user experience and have shown the usefulness for assessing user experience in virtual reality with this questionnaire. We have shown that the presence of VRISE affects the user experience, which is also in accordance with the UXIVE model of user experience in virtual environments [28]. In another study [6], we have researched the influence of video content type on users’ virtual sickness perception and physiological response. In the study, the participants watched two omnidirectional videos of two different content types on five distinct video conditions. To assess VRISE, the SSQ and SUDS, in combination with the measurement of the physiological parameters (electrodermal activity, temperature, respiratory frequency, and heart rate), were used. The results have shown that subjective questionnaires were correlated with the objective physiological measurements, whereby skin conductance strongly correlated with the VRISE levels. It was also shown that simple methods (single-scale questionnaires) could be effectively utilized to assess VRISE levels.

Weinrich et al. [12] have conducted an experiment in which they assessed user experience in VR and compared different measures for evaluating it. The study gave insights into relations between general and VR specific aspects of user experience. For the user experience, they have used the meCUE questionnaire, which is based on the components of the User Experience model [29]. The presence was assessed by iGroup Presence Questionnaire (iPQ), and for VRISE, the FMS questionnaire was used. They have also used the discomfort scale, where measured discomfort is more relevant to the ergonomic side of design, influenced by biomechanical design aspects, such as pressure points. Furthermore, the Game Experience Questionnaire (GEQ) was administered. During and after the experiment, participants rated their experience with respect to various components of general user experience as well as other components specific to VR experience. The results revealed positive correlations of presence and social presence between most of the employed post-experience UX measures.

Davis et al. [19] presented a systematic review in the area of VRISE with a focus on methods of measurement, factors, and causes that affect VRISE. They suggested that there is a need to develop more objective physiological measures of both the impact of VRISE and a person’s susceptibility to the condition.

Nesbitt et al. [30] were examining the effect of VRISE on participants’ cognitive function by measuring reaction time with a single choice Deary-Liewald Reaction Time Task. Besides measuring susceptibility to motion sickness with Motion Sickness Susceptibility Questionnaire (MSSQ) and accessing VR Sickness discomfort levels by standard Motion Sickness Assessment Questionnaire (MSAQ), they have additionally used a single-item nausea questionnaire, where subjects rated their nausea level on a subjective single-item scale recorded by participants at two-minute intervals. The nausea score was significantly correlated with both the MSSQ and MSAQ scores. They have measured a significant reduction in the reaction time due to symptoms of VR Sickness.

VRISE incidence and susceptibility during neck motion-controlled VR tasks in rehabilitation scenarios are discussed by Treleaven et al. [9]. VRISE levels were assessed by objective postural stability measurements before and after each VR module [range of motion (ROM), velocity, and accuracy]. For assessing subjective VRISE measures, the SSQ and visual analogue scale (VAS) was administered. The VAS score displayed significant positive correlations with SSQ score, change in postural stability time pre to post, ROM time, and total time. The authors found that using the VR head-mounted display (HMD) provoked VR Sickness in about one-third of the participants. Exposure time appears to be related to higher SS–VAS and should be considered in the future to limit SS when using the device.

Yamaguchi et al. [31] have in their experiment utilized the Self-Assessment Manikin (SAM) scale for emotional evaluation of the effect of displayed images on HMD in a small field of view (FOV) telescope-like virtual environment (VE). They have manipulated the FOV of a virtual camera to change the view of contents. Stronger emotional responses were observed with a smaller value of FOV. The result indicates the possibility of controlling the effectiveness of the same contents with the same HMD.

A review on VRISE and usability in VE was conducted by Musavi et al. [32]. They have argued that that the VRISE is one of the drawbacks of VE and that the usability, which is an aspect of user experience, of VR technology and system is of paramount importance in the market to attract the user. However, usability measurement of the VE is a difficult issue. They have stated that a VR interface with high usability provides the user with complete accessibility and freedom in managing and accomplishing his/her task in the VE.

Cedergen [33] has evaluated the user experience and usability of VR locomotion techniques. They have examined and compared currently prevailed locomotion techniques: joystick, teleportation, and “walk in place”. In an empirical study, they have used GEQ and System Usability Scale (SUS) to analyze and compare to determine which user experience concept was considered important and how the usability was perceived for each technique.

Pallavicini et al. [34] have researched the difference between immersive and non-immersive video games in terms of emotional response, usability, and sense of presence. Self-report questionnaires (VAS-Anxiety, VAS-Happiness, VAS-Surprise, Slater-Usoh-Steed Presence Questionnaire, and SUS) and psychophysiological measures (heart rate and skin conductance) were used to assess those factors. They have found no statistical differences between the immersive and the non-immersive condition regarding usability and performance scores. The immersive display modality was associated with higher self-reported happiness and surprise, and it also heightened the perceived sense of presence.

In the pilot study by Chen et al. [35], authors have examined if VR simulation can ensure usability and increase user satisfaction. A historical site was reconstructed, in which the participants were engaged in user experience testing. To assess the usability and user satisfaction data, the semi-structured questionnaire and non-standard 5-point scale five questions were administered after the user experience testing. The result has indicated indicates that the VR system contributed to higher satisfaction on user interactive learning and operation efficiency and thus can be used to enhance the user experience.

Hou et al. [36] developed a VR game user experience test method based on EEG signals. Subjective and objective methods are used to measure the user experience of VR games. While subjective methods are the most convenient way to assess usability, the subjective methods are more easily misguided, with eye-tracking as one of the most popular objective methods to measure usability. They have developed a new method and indicator to test virtual reality game user experience by measuring electroencephalography (EEG) signals and the brain functional connectivity (FC). The results have shown a significant difference in FC for two VR games of different usability, and a significant difference was observed in the gamma band. That indicated EEG can be a good assessment tool to analyze the user experience of VR games.

Reviewing the background and related work shows the importance of VRISE and user experience research in the field of VR. Most of the studies used standard subjective questionnaires for assessing VRISE and user experience. Assessing VRISE levels could be time consuming, and additional calculations are usually needed to get results, so our goal was to show that existing standard methods could be improved and optimized to assess VRISE levels quickly and reliably.

To the authors’ best knowledge, there is no study where VRISE and user experience were researched in such a way.

## 3. Research Questions and Hypothesis

The main research questions in this study were:

- Are questionnaires (FMS and VRNQ) besides the standard SSQ Questionnaire, suitable for accessing VRISE levels in VR? Is there a correlation between perceived VRISE assessed by the SSQ Questionnaire, FMS, and VRNQ questionnaires?
- Is a novel VRNQ questionnaire suitable for accessing user experience in Virtual Reality?
- Is VRNQ suitable to assess the acceptable quality of VR software?

We state that:

**Hypothesis** **1** **(H1).** *FMS questionnaire is suitable for accessing VRISE symptoms in VR.*

**Hypothesis** **2** **(H2).** *VRNQ (VRISE section) questionnaire is suitable for accessing VRISE symptoms in VR.*

**Hypothesis** **3** **(H3).** *VRNQ (User Experience section) is suitable for accessing user experience in VR.*

## 4. Method

This section describes the study design with the emphasis on the participants, apparatus, game design, metrics used, and experiment procedure.

### 4.1. Participants

**Figure 1:**
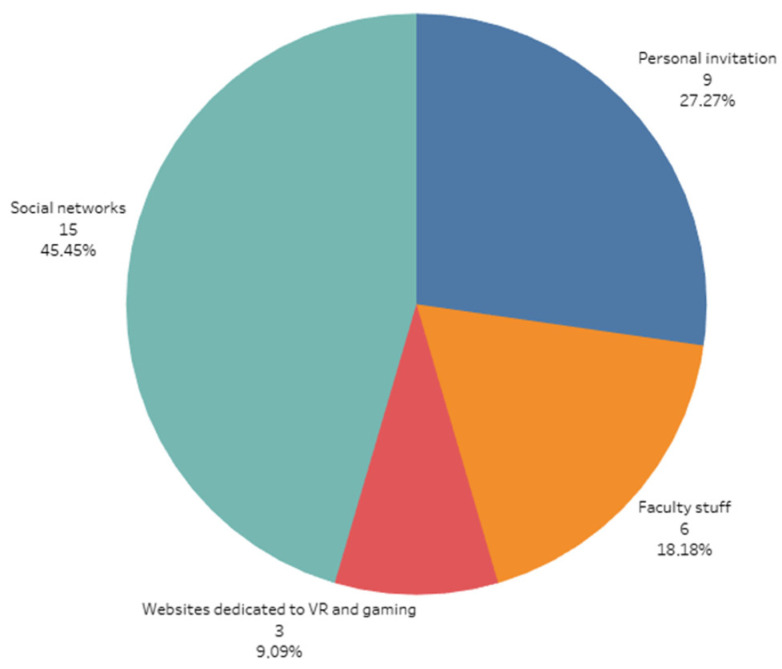
The distribution of the participants in the study based on the recruitment channel.

In the study we collected results from thirty-three participants. Twenty-five of them were males, and 8 of them were females. They ranged in age from 19 to 49 years, with a mean age of 31.97 years (SD = 8.23 years). They came from mixed backgrounds: academic staff, post-graduate researchers, students, and users interested in gaming and virtual reality technology. The participants were recruited from the Faculty of Electrical Engineering through invitations on social media and through invitations on websites dedicated to virtual reality and gaming. The distribution of the participants in the study based on the recruitment channel is shown in Figure 1. The participation was voluntary, and only healthy participants were selected.

Previous experience with VR devices had 17 participants, of which 14 of them were males, and three of them were females. Twelve of them have previously experienced side effects due to VR usage. They have reported postural instability (6), vertigo (5), sweating (5), nausea (4), disorientation (2), stomach awareness (2), headache (2), eye strain (2), dizziness (2), general discomfort (1), tiredness (1), and paleness (1).

Based on activity in the last six months, 14 of the participants were active gamers, of which 13 of them were males, and one of them was female. Eighteen participants had a corrected-to-normal vision and were wearing distance spectacles or using contact lenses. Twenty-six participants were engaged in sports, but none of them were into sports professionally. All participants provided written informed consent before taking part in the experiment and have not received any compensation for the participation.

### 4.2. Apparatus

We have used a high-performance gaming computer (CPU Intel core i7 7700K, GPU Nvidia GTX 1070, RAM 16 GB DDR4 3000 MHz, SSD Samsung Evo 850), which was connected to the Oculus Rift S head-mounted device (HMD, Oculus, Menlo Park, CA, USA). For interaction and locomotion within the virtual environment, wireless Oculus Touch controllers with 6 degrees of freedom (Lenovo) were used. Oculus Rift S features integrated inside-out tracking (without external sensors), named Oculus Insight, which was used for motion and controller tracking. The sound was played through speakers integrated into the headband of the HMD. Fulfilment of all the questionnaires by the participants was done on a notebook PC equipped with a touch screen.

### 4.3. Software and Game Design

We have developed a first-person VR game in Unity 3D [37] which the participants played for the experiment. By collecting coins placed on the predetermined path, the players had to find the way through different game scenes (forest, ancient desert, and village). To proceed into the next game scene, the players had to open the sliding doors, which divided the scenes. While developing the game, we gave extra caution that playing and interacting would be easy and intuitive even for non-gamers.

In the game, the participants had to collect the coins by passing through them, which made them disappear. To stay on the predetermined path and to not get confused about where to go forward, the next coin to collect was always visible in the virtual environment. To collect all the coins was not required to finish the level, although the participants were instructed to collect as many coins as possible. The coins were animated and were rotating around its y-axis. The participants’ virtual hands and controllers were always shown through the gameplay. The level was completed when the player passed through the finishing portal. In the game were also other action movements, such as passing through a tunnel and over a glass bridge and ascending and jumping off the building.

We have developed two different game modes, a low action (condition LA) and a high action (condition HA) game mode. Each game mode had three different conditions, without (condition NORF) or with head-centric rest-frames. The conditions with head-centric rest-frames were: glasses (condition RFG) in central vision and a baseball hat (condition RFH) in peripheral vision. 

**Figure 2:**
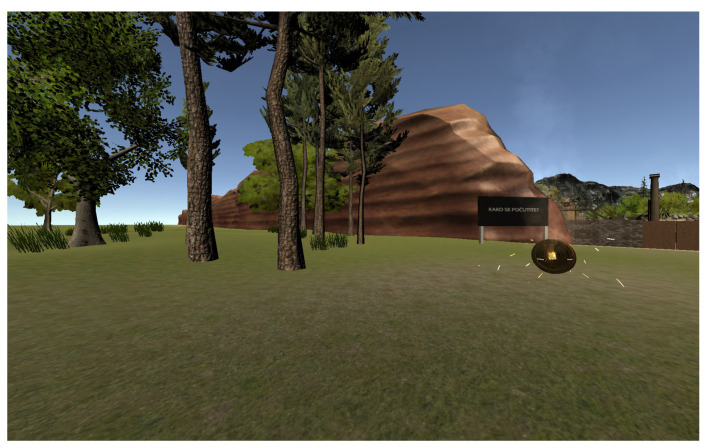
A forest scene from the low action game mode without head-centric rest-frames. On the figure, there is a visible golden coin, which is set on the player’s height. To collect the coin, the players needed to pass through it, which made the coin disappear. In the background, there is visible the big billboard, which was used to notify the players to give the FMS score. On the right part of the picture are visible doors, which was needed to be opened to proceed to the next scene of the game.

**Figure 3:**
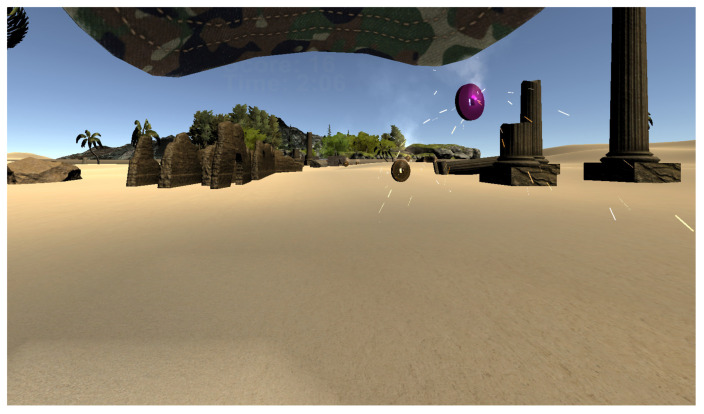
A desert scene from the high action game mode with a baseball hat as a head-centric rest-frame. On the figure, there is a visible violet coin. To collect the violet coin, the players needed to jump.

In a high action game mode, the forward-moving speed and jaw rotation speed was higher comparing to low action game mode (walking vs. running). The high action game mode also included additional provocative actions, such as jumping to collect the coins which were placed higher. A forest scene from the low action game mode without rest-frames is shown in Figure 2, and a desert scene from the high action mode is shown in Figure 3. In total there were six conditions:

- LA_NORF: low activity game mode, no rest-frames, walking, slow rotations;
- LA_RFG: low activity game mode, rest-frames glasses, walking, slow rotations;
- LA_RFH: low activity game mode, rest-frames baseball hat, walking, slow rotations;
- HA_NORF: high activity game mode, no rest-frames, running, fast rotations, jumping;
- HA_RFG: high activity game mode, rest-frames glasses, running, fast rotations, jumping;
- HA_RFH: high activity game mode, rest-frames baseball hat, running, fast rotations, jumping.

Smooth artificial locomotion was used for locomotion, a technique that is similar to the game mechanics of traditional first-person shooters (FPS) on a 2D display, where translation and jaw rotations are handled by a controller. This technique is one of the most used in VR applications. As a navigation interface, the Oculus Touch controller was used. A thumbstick on the controller was used to translate and rotate the participants’ virtual avatar. When the player was near the doors, any of the buttons on the controller (A, B, X, or Y) was used to open the doors. To perform a jump, a trigger button on the controller needed to be pressed. By physically turning their heads, the participants could freely look around. The participants were seated during gameplay.

When a coin was collected successfully, and when the door was opened, a notification sound was played via the speakers integrated into the headband of the HMD.

### 4.4. Metrics

In our study, for assessing VRISE levels, we have used the Simulator Sickness Questionnaire (SSQ) [2], Fast Motion Sickness Score (FMS) [11], and a novel Virtual Reality Neuroscience Questionnaire (VRNQ) [17]—VRISE section. For user experience, we have used the short version of the User Experience Questionnaire (UEQ-S) [16] and the VRNQ Questionnaire—User Experience section.

#### 4.4.1. VRISE

The SSQ Questionnaire is the standard measure and most commonly used in VR research for assessing VRISE. It consists of 16 items, where 16 individual symptoms of VRISE are rated with a score of 0 (none), 1 (slight), 2 (moderate), or 3 (severe). The SSQ Questionnaire provides a total SSQ score (SSQ-T) and three sub-scores: nausea (SSQ-N), disorientation (SSQ-D), and oculomotor (SSQ-O). The nausea subscale includes symptoms such as stomach awareness, increased salivation, and nausea itself. The Oculomotor subscale includes symptoms such as eyestrain, headache, and blurred vision. Disorientation includes symptoms such as dizziness, vertigo, and difficulty focusing. Higher scores indicate greater VRISE levels. SSQ questionnaire can be administered before and after the virtual experience.

FMS is a single-item verbal rating scale. To evaluate the level of sickness the respondents felt in the experience, the participants give a score from 0 (no sickness at all) to 20 (frank sickness). In contrast to the SSQ questionnaire, FMS also allows to measure participant’s VRISE levels during the experience and thus capturing its trend over time. Participants have to be focused on nausea, general discomfort, and stomach problems and take these parameters into account when making their judgments. They are asked to ignore other possible distorting effects, such as fatigue, nervousness, or boredom.

#### 4.4.2. User Experience

The UEQ-S contains eight items rated through the 7-stage Likert scale, allows a quick assessment of user experience, and measures two dimensions (quality aspects) of user experience. Pragmatic quality describes interaction qualities related to the tasks or goals the user aims to reach when using the product, and hedonic quality, which does not relate to tasks and goals but describe aspects related to pleasure or fun while using the product. 

The items on the UEQ-S questionnaire are scaled from −3 to +3. Lower scores indicate greater levels of disagreement, while higher scores indicate greater levels of agreement with scales. Thus, −3 represents the most negative answer (fully agree with the negative term), 0 a neutral answer, and +3 the most positive answer (fully agree with the positive term). All scores above value one are considered as a positive evaluation. As UEQ was already evaluated as suitable in user experience research in VR, we have decided to use the short version of the UEQ questionnaire since it allows a quick assessment and can predict the behavior of the full version concerning the pragmatic and hedonic quality of user experience. The consistency of UEQ-S scales are reasonably high [16], and the usage of UEQ-S is allowed to be used in some scenarios, such as was this experiment.

#### 4.4.3. Virtual Reality Neuroscience Questionnaire

VRNQ is a novel questionnaire dedicated to being used in VR applications. It assesses and reports both the quality of VR software attributes and VRISE intensity. VRNQ assesses and reports overall VR experience in four sections (VRISE, user experience, in-game assistance, and game mechanics). Each section has five items rated through a 7-stage Likert scale, ranging from extremely low (1) to extremely high (7). VRNQ provides a total score corresponding to the overall quality of VR software, as well as four sub-scores for each section. VRISE subscale includes items such as nausea, disorientation, dizziness, fatigue, and instability. User experience subscale includes items such as experienced immersion, enjoyment, quality of graphics, quality of sound, and overall quality of VR technology. The higher scores indicate a more positive outcome, which also applies to the evaluation of VRISE levels. It also provides the minimum and parsimonious cut-offs to appraise the suitability of VR software [17]. The minimum cut-offs indicate the lowest acceptable quality of VR software, while the parsimonious cut-offs indicate more robust VR software suitability.

In Table 1, we have compared the metrics (questionnaires) used in this study. Comparisons made are between items of questionnaires used in this study.

As shown in Table 1, the SSQ and FMS questionnaire can be executed before the experiment (to get baseline values), and after the experiment to assess the influence of the performed experiment on the participants. Measuring user experience using UEQ-S and assessing software attributes with VRNQ can only be done after the virtual experience. FMS Questionnaire is easy to understand a single-item verbal rating scale, has low complexity, and enables users to answer it quickly. SSQ and UEQ Questionnaires are more complex, have more questions, need more explanation to be given to users, and thus are slower to be answered by users. FMS also has the advantage of having results instantly after the users’ answers the questionnaire versus SSQ, UEQ-S, and VRNQ, where gathered responses needed to be calculated in an application (e.g., Excel) to get the results. For all these reasons, the FMS Questionnaire can be a very suitable method for quick assessment of VR Sickness discomfort levels. Compared VRNQ to the SSQ and FMS questionnaires, it also assesses software attributes, not just only symptoms pertinent to VRISE.

### 4.5. Experimental Environment

The experiment was performed in the Multimedia Laboratory, which was set up as a living room. Experimenters had full control over the environmental conditions, which were monitored (temperature, humidity, and lighting conditions). There were no external sources of noise that could interfere with the experiments.

### 4.6. Procedure

The experiment was performed using a 2 × 3 repeated measures within-subjects design. The participants experienced all six conditions in one session. The independent variables were: game mode (two levels: low action (LA) and high action (HA)) and simulated head-centric rest-frame (three levels: no rest-frame (NORF), rest-frame glasses (RFG), and rest-frame baseball hat (RFH)). 

Four sets of questionnaires were prepared to gather quantitative data. All questionnaires were fulfilled online via a web browser. The first set of questionnaires the participants fulfilled at home before the experiment. This questionnaire included participants’ demographic data, fitness level, vision and hearing, VR technology, and gaming experience. To be able to attend the experiment, the participant had to complete the first questionnaire. They were informed not to consume any stimulant drinks, food, or alcohol prior two hours before the experiment.

When the experiment took place, the first step was to receive the participants and to welcome them at the Multimedia Laboratory. The participants were offered to read the written document of how the experiment would be performed. We asked the participants about their possible sickness and well-being. If they were sick or had any conditions that are contraindicated for VR usage or would affect the results of the experiment, they would not be allowed to participate in the experiment. Participants then signed the informed consent for participation in the study.

After that, the participants were guided to the experimental apparatus. The experimenters helped them equip them with the Oculus Rift S and explained how to use controls, navigate, and interact in the VR game with an Oculus Touch controller. Oculus Rift S does not have the feature for the mechanical setting of interpupillary distance (IPD). It only has a software configuration setting that allows setting an IPD of a user. Therefore, we have manually measured (IPD) of the participants and configured Oculus Rift S with the measured value so that a mismatch would not contribute to VRISE.

Before playing the first game scenario, the participants played the tutorial level to get familiar with the Oculus Rift S and Oculus Touch controllers, game mechanics, and virtual environment. In this tutorial level, the participants got acquainted with navigating, jumping, rotating, collecting coins, and opening doors. We included a tutorial level in the experiment procedure so that the gameplay of experiment game scenarios would be as smooth as possible. The tutorial level session did not last as more than two minutes for any of the participants. When equipping the HMD, the participants were instructed to close their eyes and keep them closed for a few seconds after successful placement. While the game was starting, the FPS was fluctuating, so by keeping their eyes closed, the participants would not be affected by it, possibly contributing to elevated VRISE levels.

The participants were seated on a sofa during gameplay. They were using Oculus Touch controllers to translate and rotate their virtual body and were instructed to hold both Oculus Touch controllers in their hands, using one or both of them as preferred. Translating, rotating, and interacting was comfortable being right-handed or left-handed, as input controls were mirrored on both controllers. By physically turning their heads, the participants could freely look around and exploring the given scene.

After the completion of tutorial level, they were instructed to complete the second questionnaire. The participants were asked about their wellbeing and the last usage of VR technology. During the experiment, all questionnaires were fulfilled online via a web browser on a notebook PC with a touch display. 

After that, the participants began to play all six scenarios. Conditions were counterbalanced (Latin square method) across participants to account for potential order effects. Before the gameplay, the participants were not informed which condition they would play.

Immediately after finishing each game scenario, we collected participants’ feedback with the third set of questionnaires. The participants fulfilled the SSQ, UEQ-S, and VRNQ questionnaires (user experience and VRISE section). All those questionnaires were fulfilled on a computer on the other side of the room, so the participants needed to stand up and walk to the computer. We have assumed that would help them lessen VRISE levels and that the participants would be able to determine them (especially disorientation and postural instability) more precisely.

The FMS scores were collected six times for each game scenario. Before the first game scenario, the participants were instructed how to give FMS scores correctly. The first FMS score was collected before the participant has equipped with the HMD. Four FMS scores were collected at the specific locations in the virtual environment (VE) while the participants were playing the game. This was done using big billboards in VE, which displayed the text “How do you feel” when the player came near the billboard. As the FMS is a single-item verbal rating scale, the FMS score was given verbally and recorded by the researcher. The last FMS score was collected at the end of the game scenario, while the participants still had an HMD placed on the head.

Before continuing with the next game scenario, we gave participants enough time to recover from the possible side effects of the VR experience. The minimum time to proceed with the following game scenario was five minutes, but we gave the participants more time when needed. We did not allow the participants to continue with the next game scenario if the FMS collected before the gameplay was more than one, indicating negligible VRISE symptoms.

After completing all six game scenarios, the participants were instructed to complete the fourth set of questionnaires. They fulfilled the VRNQ Questionnaire (Game Mechanics and In-Game Assistance section) and NASA-TLX to determine the task load of the experiment. 

At a time, only one participant was involved in the experiment. One researcher was conducting the experiment while the other was observing and recording the FMS questionnaire’s answers. Each session lasted approximately 90 min, including introduction, signing the consent form, playing all six game scenarios, and fulfilling all the questionnaires. Active gameplay and experiencing VR game took in average 18 min and 39 s as presented in Table 2. Active gameplay or exposure time includes playing tutorial level and all the experiment scenarios. The statistically significant difference was found only between Gaming experience groups (Mann-Whitney U test, Z = −2.621, *p* = 0.009).

## 5. Results

In this section, we present the results of the study. In the analysis, we took into account only the results of the participants, which properly fulfilled all the questionnaires. Data from online questionnaires were exported into Xls files, preparation of data, calculation, and aggregation of results was done in Tableau Prep and statistically analyzed in IBM SPSS and R Studio. Spearman’s rank correlations were calculated since most of the resulting scales did not meet the requirements for the Pearson correlation coefficient.

### 5.1. VRISE

Detailed VRISE results assessed by SSQ, FMS, and VRNQ questionnaires for all conditions are presented in Table 3. Average FMS was calculated as an average score from all six verbally given scores before, during, and at the end for each game scenario.

We have observed six measured factors to find statistically significant correlations. Detailed results are shown in Table 4, Table 5, Table 6, Table 7, Table 8 and Table 9 for each condition. We have marked (greyed out) correlations that are of interest to our research and are statistically significant (*p* < 0.05).

We have calculated average VRISE scores for all conditions per user. Detailed average VRISE results assessed by SSQ, FMS, and VRNQ questionnaires are presented in Table 10.

**Figure 4:**
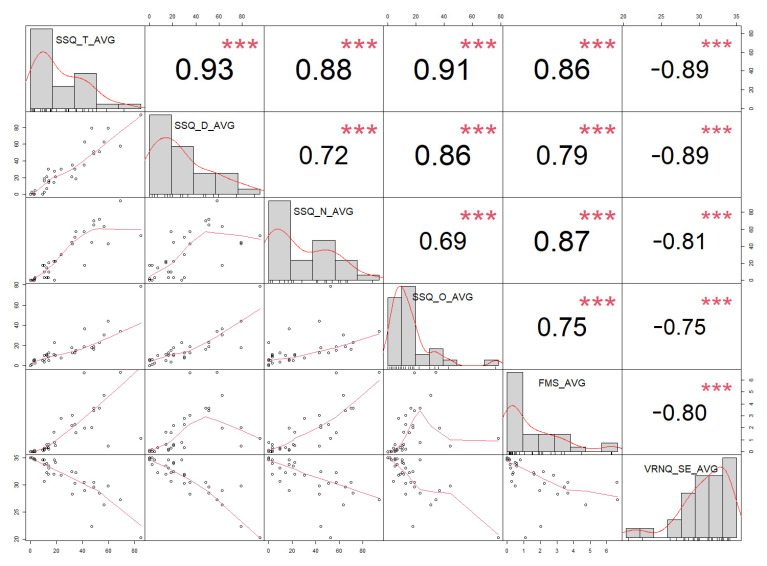
Correlation chart matrix for average VRISE scores—spearman (ρ) correlation coefficients for combined VRISE measures (*n* = 6). There are also bivariate scatterplots on the matrix, with a fitted line and histogram for each variable. * *p* < 0.05, ** *p* < 0.01, *** *p* < 0.001.

We have observed six measured factors to find statistically significant correlations. Detailed results are presented in Table 11 and Figure 4. We have marked (greyed out) correlations that are of interest for our research and are statistically significant (*p* < 0.05).

### 5.2. User Experience

Detailed UEQ_S results assessed by UEQ-S and VRNQ questionnaire are shown in Table 12.

We have observed four measured factors to find statistically significant correlations. Detailed results are shown in Table 13, Table 14, Table 15, Table 16, Table 17 and Table 18 for each condition. We have marked (greyed out) correlations that are of interest to our research and are statistically significant (*p* < 0.05).

We have calculated the average user experience scores for all conditions per user. Detailed average user experience results assessed by UEQ-S and VRNQ questionnaires are presented in Table 19.

**Figure 5:**
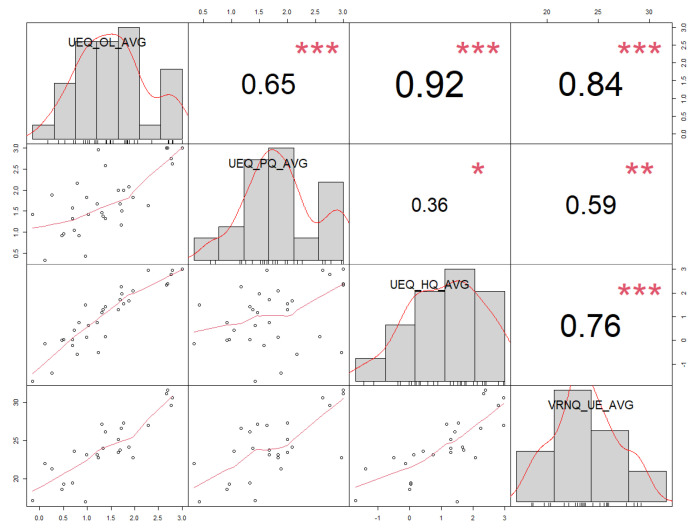
Correlation chart matrix for average user experience scores—spearman (ρ) correlation coefficients for combined user experience measures (*n* = 4). There are also bivariate scatterplots on the matrix, with a fitted line and a histogram for each variable. * *p* < 0.05, ** *p* < 0.01, *** *p* < 0.001.

We have observed six measured factors to find statistically significant correlations. Detailed results are presented in Table 20 and Figure 5. We have marked (greyed out) correlations that are of interest for our research and are statistically significant (*p* < 0.05).

### 5.3. VR Software Suitability

As VRNQ Questionnaire enables us to assess the acceptable quality of VR software, we have evaluated our game whether it meets the minimum or parsimonious cut-offs and, if it has adequate quality without any significant VRISE. The values of cut-offs are based on the median of each sub-score and totals score. The values are ≥25 for each sub-score to meet minimum cut-offs and ≥30 to meet parsimonious cut-offs. For VRNQ Total score, the value is ≥100 to meet minimum cut-offs and ≥120 to meet parsimonious cut-offs [17]. As we have administered VRNQ—VRISE subscale and VRNQ—User Experience subscale for each game scenario, we have calculated the results for each condition. VRNQ—Game Mechanics subsection and VRNQ—Game Assistance was administered at the end of all finished the six scenarios, so only one calculation was made. The results are presented in Table 21. Our game was developed in compliance with best practices to build a VR game, and extra caution was made that game was optimized to achieve a constant 80 frames per second (FPS). 80 FPS is the maximum refresh rate for the Oculus Rift S device. There was no noticeable latency of tracking during the experiment since the motion-to-photos latency is also a significant factor that affects VRISE. Because of this we have expected, that the game would meet at least the minimum cut-offs.

## 6. Discussion

### 6.1. VRISE

Analyzing correlation results for VRISE scores for each condition, as presented in Table 5, Table 6, Table 7, Table 8 and Table 9, we have observed weak to very strong statistically significant correlations between all SSQ (sub)scales and FMS scores. Moderate to very strong significant correlations were observed between all SSQ (sub)scales and VRNQ—VRISE subscale scores. Strong significant correlations were also observed between FMS and VRNQ—VRISE subscale scores. Regarding the FMS scores, the strongest correlations were observed compared to SSQ Total and SSQ Nausea scores, and weakest compared to SSQ Oculomotor scores. This was expected since the FMS Questionnaire is used to evaluate the level of sickness (nausea, general discomfort, and stomach problems) the participants felt during the virtual experience. VRNQ—VRISE subscale scores had the weakest correlations compared to SSQ Oculomotor scores, which is also expected since the VRISE subscale does not include oculomotor related items. Those results support our hypotheses H1 and H2, that the FMS and VRNQ (VRISE section) are suitable for assessing VRISE in VR. Although the correlations compared to SSQ Oculomotor subscale are statistically significant, they are less strong, so for assessing oculomotor related symptoms, the FMS and VRNQ (VRISE section) questionnaires are less suitable. VRISE symptoms profile is characterized by D > *n* > O profile, where disorientation symptoms are most severe and frequent, followed by nausea symptoms and least oculomotor symptoms [21]. Therefore using FMS and VRNQ (VRISE section) for assessing VRISE should not be an issue.

Analyzing correlation results for average VRISE scores, as presented in Table 10, we have observed strong significant correlations between all SSQ (sub)scales and FMS scores, and also strong significant correlations between all SSQ (sub)scales and VRNQ—VRISE subscale scores. Strong significant correlations were also observed between FMS and VRNQ—VRISE subscale scores. The less strong correlations were observed comparing FMS and VRNQ (VRISE section) to SSQ Disorientation scores. Those results in accordance with correlation results for each condition and additional supports our hypothesis H1 and H2 that the FMS and VRNQ (VRISE section) are suitable for assessing VRISE in VR.

### 6.2. User Experience

Analyzing correlation results for user experience scores for each condition, as presented in Table 13, Table 14, Table 15, Table 16, Table 17 and Table 18, we have observed weak to very strong statistically significant correlations between all UEQ-S (sub)scale scores and VRNQ—User Experience subscale scores. The only exception was for the LA_NORF condition, where the correlation between UEQ-S Pragmatic Quality scores and VRNQ—User Experience subscale scores was not significant. For other conditions, the correlations between UEQ-S Pragmatic Quality scores and VRNQ—User Experience subscale scores were statistically significant, although they were less strong (weak to strong). Because VRNQ (User experience section) questionnaire items are more related to hedonic aspects of user experience (immersion, enjoyment, quality of graphics, quality of sound, and overall quality of VR technology), and because of less strong correlations compared to UEQ-S Pragmatic Quality scores, the VRNQ is less suitable to access the pragmatic quality of user experience. However, the results support our hypothesis H3 that the VRNQ (User experience section) is suitable for assessing user experience in VR.

Analyzing correlation results for average user experience scores, as presented in Table 20, we have observed weak and strong significant correlations between all UEQ-S (sub)scales and VRNQ—VRISE user experience scores. Strong significant correlations were observed between UEQ-S Overall and UEQ-S Hedonic Quality compared to VRNQ (User Experience section), and weak between UEQ-S Pragmatic Quality and VRNQ (User Experience section). Those results are in accordance with correlation results for each condition and additional supports our hypothesis H3 that the VRNQ (User Experience section) is suitable for assessing user experience in VR.

### 6.3. VR Software Suitability

Based on VRNQ for assessing adequate VR software quality, as presented in Table 21, the game had adequate quality in terms of VRISE for each condition and also for average VRISE scores, exceeding parsimonious cut-off criteria. It also had adequate quality in terms of Game Assistance, exceeding minimum cut-off criteria. 

The game had not adequate quality in terms of user experience for each condition and average user experience scores, not exceeding minimum cut-off criteria. That was expected and not relevant for our game because one of the items of VRNQ (User Experience section) is about the quality of sound. Not meeting minimum cut-off levels for user experience was due to almost no existent sound or ambient music in the game, which was not emphasized in the game. Making sound more expressed, the game would easily meet adequate VR software quality in terms of user experience since medians of VRNQ (User Experience section) scores were near cut-off values.

The game had not adequate quality in terms of Game Mechanics, not exceeding minimum cut-off criteria. That was expected and not relevant for our game because one of the items of VRNQ (Game Mechanics section) is about the availability of physical movement (room-scale locomotion). Because of the experiment’s design, room-scale locomotion was not used. We have used the seated VR experience. If the game would be played with room-scale locomotion, the game would easily meet adequate VR software quality in terms of game mechanics since medians of VRNQ (Game Mechanics section) scores were near cut-off values.

## 7. Limitations and Mitigations

Using the short version of the User Experience Questionnaire (UEQ-S) as a basis for correlation results could be argued. UEQ-S measures just two dimensions (quality aspects) of user experience (hedonic and pragmatic quality), and the consistency of those scales are reasonably high [16]. UEQ-S does not cover all six aspects of user experience, as the standard UEQ Questionnaire [27] does. However, VRNQ—VRISE subscale is also simplified and includes items such as immersion, enjoyment, quality of the graphics, quality of the sound, quality of the VR technology. As UEQ has already been proven to be useful in VR research [7], and UEQ-S can predict the behavior of the full version concerning pragmatic and hedonic quality, the selection of UEQ-S was appropriable. Also, the usage of UEQ-S is allowed to be used in some scenarios, such as was this experiment.

We have decided to collect six FMS verbal rated scores for each game scenario. The timing, location of collections in the virtual environment, and the total number of collections could be argued. But given that, the FMS was collected before and after each game scenario and after finishing each of the game scenes in the game, the timing, location, and the total number of FMS collections seems appropriate.

## 8. Conclusions and Future Work

Despite the already achieved technological maturity of virtual reality technologies (VR), the health-oriented side effects are still common between users of this new technology, which can negatively impact the broader technology adoption. In the last five years, a lot of VR devices and technology solutions were introduced. Plenty of different VR applications, dedicated for home and professional use, have been released. There are several interactions and locomotion techniques, which can also affect the impact of technology on users. Besides health-oriented side effects that come with the VR usage, the quality of user experience also has an important impact on the user adoption and success of VR. Assessing user experience in VR is possible with several questionnaires, but none of them are widely adopted yet. Thus, there is a need for standard, reliable, and quick assessment tools that should be developed, especially for VR applications. Their aim should be focused to assess and compare different technology solutions and to assess the impact of different interaction and locomotion techniques on the users in terms of Virtual Reality-Induced Symptoms and Effects (VRISE) and user experience. Assessing VRISE levels and user experience could be time consuming, especially when using objective physiological measures

In our study, we have presented the results of the suitability and comparison of questionnaires assessing VRISE and user experience in virtual environments. For VRISE, we have compared the standard SSQ Questionnaire with FMS and a novel VRNQ Questionnaire (VRISE section). For user experience, we have compared the short version of the UEQ Questionnaire (UEQ-S) and a novel VRNQ Questionnaire (User Experience section). SSQ is widely used in VRISE studies. To a lesser extent, is the UEQ Questionnaire used in studies of quality of user experience in VR. The problem with both questionnaires (SSQ and UEQ) is that they were not developed for usage in VR. Our goal was to show that existing standard methods for assessing VRISE and user experience could be replaced with improved and optimized methods for evaluating VRISE and user experience.

The key advantage of both FMS and VRNQ is that they are shorter, easier to understand, and especially faster to fulfill. VRNQ provides a total score corresponding to the overall quality of VR software, as well as four sub-scores for each section/domain (User Experience, Game Mechanics, In-Game Assistance, and VRISE). In our study, user experience and VRISE sections were of importance. FMS is a single-item verbal rating scale and also enables to record the VRISE levels during the virtual experience and capture its time course. FMS have also the advantage of having results instantly after the users answer the questionnaire. Another advantage of VRNQ is that it is dedicated for usage in VR applications, and it also give us information if evaluated VR software have an adequate quality without any significant VRISE.

The results have shown that FMS and a novel VRNQ Questionnaire (VRISE section) are suitable for assessing VRISE levels in VR. The results have also shown that a novel VRNQ Questionnaire (User Experience section) is suitable for assessing the quality of user experience in VR. As VRNQ also enables us to appraise VR software’s suitability, we have also evaluated the adequate software quality of our game.

The contribution of this study is important towards the goal to achieve the standard, reliable, and quick objective measurement tools for VR. In future works, the presented questionnaires should be tested for some other VR content types, such as 360 videos and room-scale experiences. In our study, the questionnaires were tested in a gaming content in a seating experience. We have put the focus on VRISE and user experience, and since VRNQ also assesses in-game assistance and game mechanics of VR experience, those two subscales should also be more thoroughly analyzed for their suitability in future studies. However, we found VRNQ suitable for assessing the quality of VR software attributes and VR software suitability.

In future studies, we encourage the usage of Fast Motion Sickness Score (FMS) for assessing VRISE, and Virtual Reality Neuroscience Questionnaire (VRNQ) for assessing user experience, in-game assistance, and game mechanics, besides VRISE. Both of the questionnaires were found suitable, and they are easy to administer and understand. FMS also enables to record the VRISE levels during the virtual experience and thus capturing its trend over time. Another yet important advantage of the FMS Questionnaire is that no additional calculation is needed to get the results and asses VRISE levels.

## Figures and Tables

**Table 1:** Comparison of used metrics (questionnaires) in the study.

	Simulator Sickness Questionnaire (SSQ)	Fast Motion Sickness Score (FMS)	Short Version of the User Experience Questionnaire (UEQ-S)	Virtual Reality Neuroscience Questionnaire (VRNQ)
Type	Subjective Questionnaire	A single-item verbal rating scale	Subjective Questionnaire	Subjective Questionnaire
Purpose	Assessing VRISE	Assessing nausea related VRISE symptoms	Assessing user experience	Assessing VRISE, user experience, game mechanics and in-game assistance
Number of Questions	16	1	8	20
Scoring	0 (none), 1 (slight), 2 (moderate), 3 (severe)	from 0 to 20	7-stage Likert scale (scored from −3 to +3)	7-stage Likert scale (scored from 1 to 7)
Sub-scores	Yes: Disorientation (SSQ-D), Nausea (SSQ-N), Oculomotor (SSQ-O)	No	Yes: Pragmatic and Hedonic Quality of user experience	Yes: User Experience, Game mechanics, In-game assistance, VRISE
Execution	Before and after scenario	Before, during, and after scenario	After scenario	After scenario
Administration time	Medium (e.g., 5 min)	Very fast (e.g., 15 s)	Fast (1 min)	Medium (e.g., 2–5 min)
Complexity	Medium	Very low	Medium	Low
Additional calculation needed to get results	Yes	No	Yes	Yes
Suitable for quick assessment	No	Yes	No	No

**Table 2:** Exposure time in virtual reality (VR), including tutorial level. The data presented is for all participants and subgroups based on gender, previous gaming and VR experience.

(sub)Group	N	Mean	Minimum	Maximum	SD
All participants	32	18 min 39 s	14 min 21 s	27 min 17 s	3 min 13 s
Gender—males	24	18 min 15 s	14 min 21 s	25 min 32 s	3 min 7 s
Gender—females	8	19 min 52 s	15 min 53 s	27 min 17 s	3 min 25 s
Gaming experience—Yes	14	16 min 55 s	14 min 42 s	20 min 29 s	1 min 44 s
Gaming experience—No	18	20 min 0 s	14 min 21 s	27 min 17 s	3 min 30 s
VR experience—Yes	16	18 min 13 s	14 min 21 s	23 min 21 s	2 min 19 s
VR experience—No	16	19 min 5 s	14 min 42 s	27 min 17 s	3 min 57 s

**Table 3:** Subjective VRISE levels assessed by SSQ, FMS, and VRNQ (VRISE Section) questionnaire. The results are presented by game conditions.

VRISE Scale	Condition	N	Mean	SD
SSQ Total	LA_NORF	33	21.78	23.04
	LA_RFG	33	27.31	26.70
	LA_RFH	33	30.49	30.49
	HA_NORF	33	27.88	27.75
	HA_RFG	33	27.77	25.11
	HA_RFH	33	25.84	22.74
SSQ	LA_NORF	33	21.93	29.33
Disorientation	LA_RFG	33	31.21	35.32
	LA_RFH	33	32.90	36.96
	HA_NORF	33	31.64	34.15
	HA_RFG	33	27.42	28.16
	HA_RFH	33	27.84	27.18
SSQ Nausea	LA_NORF	33	23.99	24.45
	LA_RFG	33	26.60	26.38
	LA_RFH	33	32.96	35.38
	HA_NORF	33	30.35	34.76
	HA_RFG	33	31.22	33.58
	HA_RFH	33	28.04	30.35
SSQ	LA_NORF	33	13.32	17.98
Oculomotor	LA_RFG	33	17.23	19.81
	LA_RFH	33	17.69	19.11
	HA_NORF	33	15.16	16.19
	HA_RFG	33	16.54	16.78
	HA_RFH	33	14.93	17.00
FMS	LA_NORF	33	1.25	1.81
Average	LA_RFG	33	1.15	1.52
	LA_RFH	33	1.46	1.86
	HA_NORF	33	1.94	2.76
	HA_RFG	33	1.98	2.71
	HA_RFH	33	1.66	2.03
VRNQ	LA_NORF	33	32.00	3.73
VRISE scale	LA_RFG	32	31.28	4.42
	LA_RFH	33	31.97	3.32
	HA_NORF	33	30.97	4.34
	HA_RFG	33	31.03	4.57
	HA_RFH	33	31.33	3.70

**Table 4:** Correlation matrix for LA_NORF condition—Spearman (ρ) correlation coefficients for combined VRISE measures (*n* = 6).

Variables		1.	2.	3.	4.	5.	6.
1.	SSQ Total	1.000					
2.	SSQ Disorientation	0.878 **	1.000				
3.	SSQ Nausea	0.873 **	0.630 **	1.000			
4.	SSQ Oculomotor	0.903 **	0.829 **	0.632 **	1.000		
5.	FMS Average	0.786 **	0.673 **	0.797 **	0.550 **	1.000	
6.	VRNQ—VRISE subscale	−0.780 **	−0.638 **	−0.819 **	−0.637 **	−0.734 **	1.000

** *p* < 0.01 (2-tailed significance).

**Table 5:** Correlation matrix for LA_RFG condition—Spearman (ρ) correlation coefficients for combined VRISE measures (*n* = 6).

Variables		1.	2.	3.	4.	5.	6.
1.	SSQ Total	1.000					
2.	SSQ Disorientation	0.881 **	1.000				
3.	SSQ Nausea	0.881 **	0.654 **	1.000			
4.	SSQ Oculomotor	0.873 **	0.707 **	0.663 **	1.000		
5.	FMS Average	0.671 **	0.611 **	0.774 **	0.396 *	1.000	
6.	VRNQ—VRISE subscale	−0.788 **	−0.783 **	−0.760 **	−0.514 **	−0.739 **	1.000

* *p* < 0.05; ** *p* < 0.01 (2-tailed significance).

**Table 6:** Correlation matrix for LA_RFH condition—Spearman (ρ) correlation coefficients for combined VRISE measures (*n* = 6).

Variables		1.	2.	3.	4.	5.	6.
1.	SSQ Total	1.000					
2.	SSQ Disorientation	0.905 **	1.000				
3.	SSQ Nausea	0.881 **	0.675 **	1.000			
4.	SSQ Oculomotor	0.924 **	0.871 **	0.693 **	1.000		
5.	FMS Average	0.789 **	0.666 **	0.788 **	0.659 **	1.000	
6.	VRNQ—VRISE subscale	−0.816 **	−0.761 **	−0.774 **	−0.714 **	−0.689 **	1.000

** *p* < 0.01 (2-tailed significance).

**Table 7:** Correlation matrix for HA_NORF condition—Spearman (ρ) correlation coefficients for combined VRISE measures (*n* = 6).

Variables		1.	2.	3.	4.	5.	6.
1.	SSQ Total	1.000					
2.	SSQ Disorientation	0.862 **	1.000				
3.	SSQ Nausea	0.908 **	0.696 **	1.000			
4.	SSQ Oculomotor	0.842 **	0.604 **	0.686 **	1.000		
5.	FMS Average	0.831 **	0.759 **	0.807 **	0.653 **	1.000	
6.	VRNQ—VRISE subscale	−0.861 **	−0.867 **	−0.714 **	0.661 **	−0.691 **	1.000

** *p* < 0.01 (2-tailed significance).

**Table 8:** Correlation matrix for HA_RFG condition—Spearman (ρ) correlation coefficients for combined VRISE measures (*n* = 6).

Variables		1.	2.	3.	4.	5.	6.
1.	SSQ Total	1.000					
2.	SSQ Disorientation	0.918 **	1.000				
3.	SSQ Nausea	0.899 **	0.768 **	1.000			
4.	SSQ Oculomotor	0.877 **	0.809 **	0.635 **	1.000		
5.	FMS Average	0.835 **	0.790 **	0.850 **	0.675 **	1.000	
6.	VRNQ—VRISE subscale	−0.863 **	−0.867 **	−0.778 **	−0.737 **	−0.770 **	1.000

** *p* < 0.01 (2-tailed significance).

**Table 9:** Correlation matrix for HA_RFH condition—Spearman (ρ) correlation coefficients for combined VRISE measures (*n* = 6).

Variables		1.	2.	3.	4.	5.	6.
1.	SSQ Total	1.000					
2.	SSQ Disorientation	0.930 **	1.000				
3.	SSQ Nausea	0.878 **	0.721 **	1.000			
4.	SSQ Oculomotor	0.872 **	0.862 **	0.589 **	1.000		
5.	FMS Average	0.845 **	0.785 **	0.849 **	0.611 **	1.000	
6.	VRNQ—VRISE subscale	−0.861 **	−0.888 **	−0.760 **	−0.683 **	−0.794 **	1.000

** *p* < 0.01 (2-tailed significance).

**Table 10:** Subjective average VRISE levels assessed by SSQ, FMS, and VRNQ (VRISE Section) Questionnaire.

VRISE Scale	N	Mean	SD	Min Value	Max Value
SSQ Total	33	26.86	22.09	0.00	84.77
SSQ Disorientation	33	28.82	26.09	0.00	95.12
SSQ Nausea	33	28.86	26.72	0.00	93.81
SSQ Oculomotor	33	15.81	15.34	0.00	78.33
FMS Average	33	1.57	1.85	0.00	6.64
VRNQ—VRISE subscale	32	31.36	3.61	20.33	35.00

**Table 11:** Correlation matrix for average VRISE scores—spearman (ρ) correlation coefficients for combined measures (*n* = 6).

Variables		1.	2.	3.	4.	5.	6.
1.	SSQ Total	1.000					
2.	SSQ Disorientation	0.928 **	1.000				
3.	SSQ Nausea	0.882 **	0.723 **	1.000			
4.	SSQ Oculomotor	0.912 **	0.861 **	0.687 **	1.000		
5.	FMS Average	0.865 **	0.790 **	0.868 **	0.754 **	1.000	
6.	VRNQ—VRISE subscale	−0.885 **	−0.890 **	−0.805 **	−0.750 **	−0.798 **	1.000

** *p* < 0.01 (2-tailed significance).

**Table 12:** Subjective user experience levels assessed by UEQ-S and VRNQ (User Experience Section) Questionnaire. The results are presented by game conditions.

User Experience Scale	Condition	N	Mean	SD
UEQ-S	LA_NORF	33	1.37	0.96
Overall	LA_RFG	33	1.13	1.18
	LA_RFH	33	1.30	1.02
	HA_NORF	33	1.62	0.74
	HA_RFG	33	1.53	0.92
	HA_RFH	33	1.39	1.00
UEQ-S	LA_NORF	33	2.03	0.77
Pragmatic	LA_RFG	33	1.48	1.15
Quality	LA_RFH	33	1.69	0.95
	HA_NORF	33	1.82	0.84
	HA_RFG	33	1.79	0.97
	HA_RFH	33	1.71	0.97
UEQ-S	LA_NORF	33	0.70	1.58
Hedonic	LA_RFG	33	0.79	1.57
Quality	LA_RFH	33	0.92	1.47
	HA_NORF	33	1.43	1.06
	HA_RFG	33	1.27	1.28
	HA_RFH	33	1.08	1.4
VRNQ	LA_NORF	32	23.31	3.98
User	LA_RFG	31	23.00	3.86
Experience	LA_RFH	33	22.76	4.06
scale	HA_NORF	32	23.59	4.62
	HA_RFG	30	24.37	4.57
	HA_RFH	33	24.12	4.52

**Table 13:** Correlation matrix for LA_NORF condition—Spearman (ρ) correlation coefficients for combined user experience measures (*n* = 4).

Variables		1.	2.	3.	4.
1.	UEQ Overall	1.000			
2.	UEQ Pragmatic Quality	0.518 **	1.000		
3.	UEQ Hedonic Quality	0.914 **	0.182	1.000	
4.	VRNQ—User Experience subscale	0.700 **	0.310	0.628 **	1.000

** *p* < 0.01 (2-tailed significance).

**Table 14:** Correlation matrix for LA_RFG condition—Spearman (ρ) correlation coefficients for combined user experience measures (*n* = 4).

Variables		1.	2.	3.	4.
1.	UEQ Overall	1.000			
2.	UEQ Pragmatic Quality	0.757 **	1.000		
3.	UEQ Hedonic Quality	0.909 **	0.449 **	1.000	
4.	VRNQ—User Experience subscale	0.684 **	0.445 *	0.646 **	1.000

* *p* < 0.05; ** *p* < 0.01 (2-tailed significance).

**Table 15:** Correlation matrix for LA_RFH condition—Spearman (ρ) correlation coefficients for combined user experience measures (*n* = 4).

Variables		1.	2.	3.	4.
1.	UEQ Overall	1.000			
2.	UEQ Pragmatic Quality	0.659 **	1.000		
3.	UEQ Hedonic Quality	0.925 **	0.414 *	1.000	
4.	VRNQ—User Experience subscale	0.726 **	0.491 **	0.697 **	1.000

* *p* < 0.05; ** *p* < 0.01 (2-tailed significance).

**Table 16:** Correlation matrix for HA_NORF condition—Spearman (ρ) correlation coefficients for combined user experience measures (*n* = 4).

Variables		1.	2.	3.	4.
1.	UEQ Overall	1.000			
2.	UEQ Pragmatic Quality	0.656 **	1.000		
3.	UEQ Hedonic Quality	0.873 **	0.267	1.000	
4.	VRNQ—User Experience subscale	0.561 **	0.371 *	0.538 **	1.000

* *p* < 0.05; ** *p* < 0.01 (2-tailed significance).

**Table 17:** Correlation matrix for HA_RFG condition—Spearman (ρ) correlation coefficients for combined user experience measures (*n* = 4).

Variables		1.	2.	3.	4.
1.	UEQ Overall	1.000			
2.	UEQ Pragmatic Quality	0.741 **	1.000		
3.	UEQ Hedonic Quality	0.875 **	0.384 *	1.000	
4.	VRNQ—User Experience subscale	0.879 **	0.722 **	0.757 **	1.000

* *p* < 0.05; ** *p* < 0.01 (2-tailed significance).

**Table 18:** Correlation matrix for HA_RFH condition—Spearman (ρ) correlation coefficients for combined user experience measures (*n* = 4).

Variables		1.	2.	3.	4.
1.	UEQ Overall	1.000			
2.	UEQ Pragmatic Quality	0.754 **	1.000		
3.	UEQ Hedonic Quality	0.676 **	0.257	1.000	
4.	VRNQ—User Experience subscale	0.749 **	0.541 **	0.549 **	1.000

** *p* < 0.01 (2-tailed significance).

**Table 19:** Subjective average user experience scores assessed by UEQ-S and VRNQ (User Experience Section) Questionnaire.

User Experience Scale	N	Mean	SD	Min Value	Max Value
UEQ-S Overall	33	1.39	0.82	−0.15	3.00
UEQ-S Pragmatic Quality	33	1.75	0.72	0.33	3.00
UEQ-S Hedonic Quality	33	1.03	1.24	−1.71	3.00
VRNQ—User Experience subscale	33	24.19	4.06	17.00	31.67

**Table 20:** Correlation matrix for average User Experience scores—Spearman (ρ) correlation coefficients for combined measures (*n* = 4).

Variables		1.	2.	3.	4.
**1.**	UEQ Overall	1.000			
**2.**	UEQ Pragmatic Quality	0.652 **	1.000		
**3.**	UEQ Hedonic Quality	0.921 **	0.356 *	1.000	
**4.**	VRNQ—User Experience subscale	0.843 **	0.594 **	0.764 **	1.000

* *p* < 0.05; ** *p* < 0.01 (2-tailed significance).

**Table 21:** Adequate quality of VR software assessed by VRNQ Questionnaire. The results are presented by game conditions for user experience and VRISE subscales of VRNQ Questionnaire, and also for average scores across all six conditions. As the Game Mechanics and Game Assistance part of the VRNQ Questionnaire were fulfilled only at the end of the experiment, the results are not presented by game conditions. The results for the VRISE subscale of the VRNQ Questionnaire are also given for average scores across high activity and low activity modes of the game. Since cut-offs for assessing the adequate quality of VR software are based on the values of the medians, the medians are included in the descriptive statistics.

VRNQ Scale	Condition	N	Mean	Median	Min Value	Max Value
User	LA_NORF	32	23.31	22.50	18	33
Experience	LA_RFG	31	23.00	24.00	16	30
	LA_RFH	33	22.76	23.00	13	31
	HA_NORF	32	23.59	23.50	14	33
	HA_RFG	30	24.37	24.00	18	33
	HA_RFH	33	24.12	24.00	14	33
VRISE	LA_NORF	33	32.00	33.00	20	35
	LA_RFG	32	31.28	32.50	15	35
	LA_RFH	33	31.97	33.00	21	35
	HA_NORF	33	30.97	32.00	20	35
	HA_RFG	33	31.03	33.00	19	35
	HA_RFH	33	31.33	32.00	22	35
User Experience	Average	26	24.19	23.75	17.00	31.67
VRISE	Average	32	31.36	32.17	20.33	35.00
Game Mechanics	All scenarios	33	24.09	24.00	16	32
Game Assistance	All scenarios	33	27.09	26.00	15	35
VRISE	HA Average	33	31.11	32.00	20.67	35
VRISE	LA Average	33	32.00	33.00	20	35

## Data Availability

The data presented in this study are available on request from the corresponding author.
